# *catena*-Poly[[silver(I)-μ-*N*-[(pyridin-2-yl)meth­yl]pyridine-3-amine-κ^2^ *N*:*N*′] hexa­fluorido­phosphate]

**DOI:** 10.1107/S1600536814011465

**Published:** 2014-05-24

**Authors:** Suk-Hee Moon, Ki-Min Park

**Affiliations:** a Department of Food & Nutrition, Kyungnam College of Information and Technology, Busan 617-701, Republic of Korea; b Research Institute of Natural Sciences, Gyeongsang National University, Jinju 660-701, Republic of Korea

## Abstract

In the title polymeric complex, {[Ag(C_11_H_11_N_3_)]PF_6_}_*n*_, the Ag^I^ ion is two-coordinated in a nearly linear coordination geometry [N—Ag—N = 175.98 (9)°] by two pyridine N atoms from two symmetry-related *N*-[(pyridine-2-yl)meth­yl]pyridine-3-amine ligands. Each Ag^I^ ion is bridged by the ligands, forming a helical chain propagating along the *b-*axis direction. The right- and left-handed helical chains are alternately arranged *via* Ag⋯Ag [3.2639 (5) Å] and π–π stacking inter­actions [centroid–centroid distance = 3.523 (1) Å], resulting in the formation of a two-dimensional supra­molecular network extending parallel to (101). Weak Ag⋯F inter­actions [longest Ag⋯F inter­action = 3.153 (2) Å], as well as N—H⋯F and C—H⋯F hydrogen-bonding inter­actions, occur between the helical chains and the anions.

## Related literature   

For structures of Ag^I^ coordination polymers with symmetrical dipyridyl ligands, see: Lee *et al.* (2012 ▶); Leong & Vittal (2011 ▶); Park *et al.* (2010 ▶) and of Ag^I^ coordination polymers with unsymmetrical dipyridyl ligands, see: Moon & Park (2013 ▶); Zhang *et al.* (2013 ▶). For the synthesis of the ligand, see: Lee *et al.* (2013 ▶).
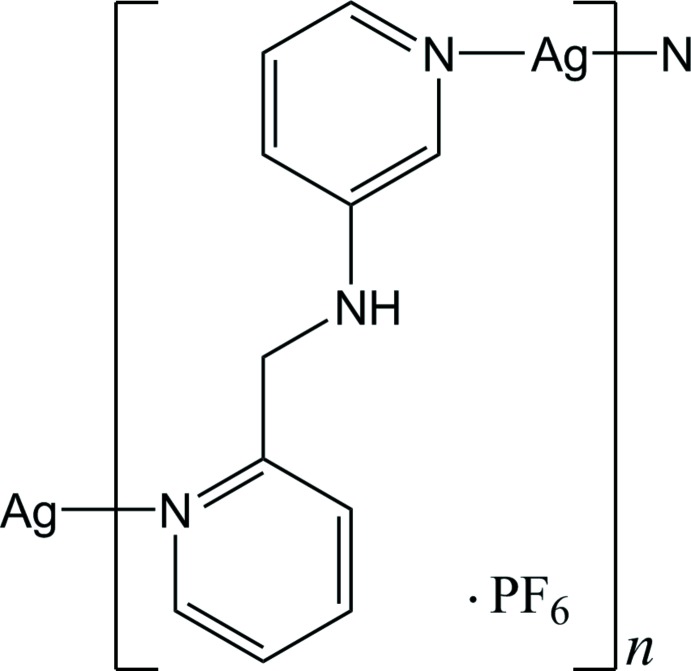


## Experimental   

### 

#### Crystal data  

[Ag(C_11_H_11_N_3_)]PF_6_
*M* *_r_* = 438.07
Monoclinic, 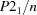

*a* = 10.9978 (6) Å
*b* = 10.5081 (6) Å
*c* = 12.7559 (7) Å
β = 108.976 (1)°
*V* = 1394.04 (13) Å^3^
*Z* = 4
Mo *K*α radiation
μ = 1.63 mm^−1^
*T* = 173 K
0.25 × 0.25 × 0.20 mm

#### Data collection  

Bruker SMART CCD area-detector diffractometer
Absorption correction: multi-scan (*SADABS*; Bruker, 2000 ▶) *T*_min_ = 0.687, *T*_max_ = 0.737
7790 measured reflections
2740 independent reflections
2509 reflections with *I* > 2σ(*I*)
*R*_int_ = 0.018

#### Refinement  

*R*[*F* ^2^ > 2σ(*F* ^2^)] = 0.027
*wR*(*F* ^2^) = 0.071
*S* = 1.05
2740 reflections
199 parameters
H-atom parameters constrained
Δρ_max_ = 0.86 e Å^−3^
Δρ_min_ = −0.44 e Å^−3^

### 

Data collection: *SMART* (Bruker, 2000 ▶); cell refinement: *SAINT-Plus* (Bruker, 2000 ▶); data reduction: *SAINT-Plus*; program(s) used to solve structure: *SHELXS97* (Sheldrick, 2008 ▶); program(s) used to refine structure: *SHELXL97* (Sheldrick, 2008 ▶); molecular graphics: *DIAMOND* (Brandenburg, 2005 ▶); software used to prepare material for publication: *SHELXTL* (Sheldrick, 2008 ▶).

## Supplementary Material

Crystal structure: contains datablock(s) I, New_Global_Publ_Block. DOI: 10.1107/S1600536814011465/sj5402sup1.cif

Structure factors: contains datablock(s) I. DOI: 10.1107/S1600536814011465/sj5402Isup2.hkl

CCDC reference: 1003661

Additional supporting information:  crystallographic information; 3D view; checkCIF report

## Figures and Tables

**Table 1:** Hydrogen-bond geometry (Å, °)

*D*—H⋯*A*	*D*—H	H⋯*A*	*D*⋯*A*	*D*—H⋯*A*
N2—H2⋯F5^i^	0.88	2.51	3.354 (4)	160
C4—H4⋯F1^ii^	0.95	2.52	3.336 (4)	145
C5—H5⋯F5^iii^	0.95	2.55	3.447 (4)	157
C5—H5⋯F6^iii^	0.95	2.43	3.280 (4)	149
C6—H6*A*⋯F3^iv^	0.99	2.54	3.467 (4)	155
C11—H11⋯F3^v^	0.95	2.49	3.397 (4)	160

Symmetry codes: (i) 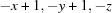; (ii) 
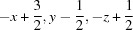
; (iii) 
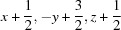
; (iv) 
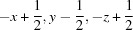
; (v) 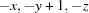.

**Fig. 1.**
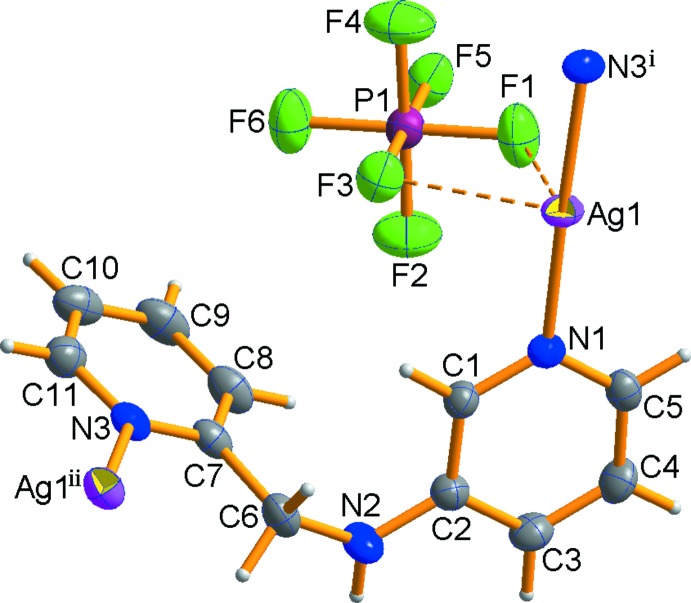
A view of the molecular structure of the title compound, with atom numbering. Displacement ellipsoids are drawn at the 50% probability level and dashed lines present Ag···F contacts [Symmetry codes: (i) 1/2 - x, 1/2 + y, 1/2 - z; (ii) 1/2 - x, -1/2 + y, 1/2 - z].

**Fig. 2.**
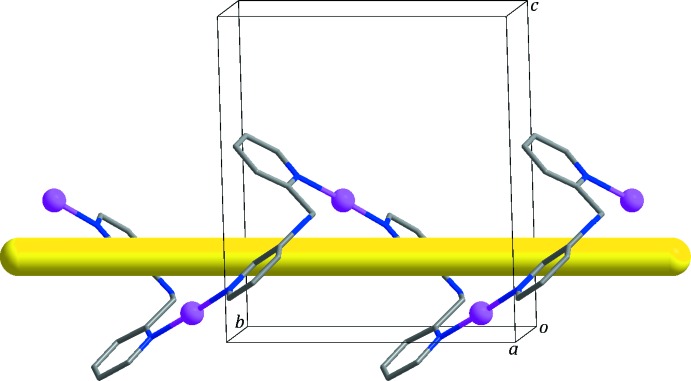
The helical chain formed by cationic polymer of the title compound along the b axis.

**Fig. 3.**
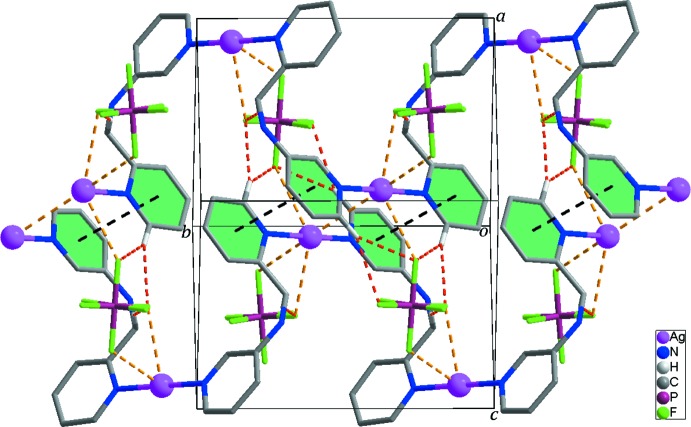
The two-dimensional supramolecular structure formed through Ag···Ag and Ag···F interactions (yellow dashed lines) and π–π stacking interactions (black dashed lines). Red dashed lines present N–H···F hydrogen bonds.

## supplementary crystallographic information

### 1. Comment

Metal-organic frameworks based on silver ions and dipyridyl type ligands have been of increasing interest in coordination chemistry owing to their intriguing architectures caused by the variety of coordination geometries of the Ag(I) ions (Lee *et al.*, 2012; Leong & Vittal, 2011; Park *et al.*, 2010). Recently, we have reported an investigation of Ag(I) coordination polymers using unsymmetrical dipyridyl ligands with nitrogen donor atoms in different positions on the two terminal pyridines (Moon & Park, 2013; Zhang *et al.*, 2013). In extending this work, *N*-(pyridine-2-ylmethyl)pyridine-3-amine as an unsymmetrical dipyridyl ligand was prepared by the reaction of 3-aminopyridine and 2-pyridinecarboxaldehyde according to a previously reported method (Lee *et al.*, 2013). Herein we report the crystal structure of the title compound prepared by the reaction of silver hexafluorophosphate with the unsymmetrical dipyridyl ligand. The structure of the title compound is isostructural with that of the perchlorate salt (Zhang *et al.*, 2013).

The title compound is shown in Fig. 1. The asymmetric unit contains one Ag^I^ cation, one *N*-(pyridine-2-ylmethyl)pyridine-3-amine (Lee *et al.*, 2013) ligand and one PF_6_^-^ anion. The Ag atom links two pyridine N atoms from two symmetry-related ligands to form a helical chain. Thus the Ag atom is two-coordinate in a slightly distorted linear coordination geometry [N–Ag–N = 175.98 (9)°]. The helical chain with a pitch length of 10.5081 (6) Å propagates along the *b* axis (Fig. 2). Right- and left-handed helical chains are alternately arranged *via* Ag···Ag [3.2639 (5) Å] and π–π stacking interactions [centroid-centroid distance = 3.523 (1) Å] between pyridine rings of the helical chains, resulting in the formation of a two-dimensional supramolecular network extending parallel to the (101) plane (Fig. 3).

The non-coordinating PF_6_^-^ anions participate in Ag···F interactions (Ag1···F1 3.035 (2), Ag1···F3 3.041 (2), Ag1···F6^iii^ 3.153 (2) Å, symmetry code: (iii) 1/2 + *x*, 1.5 - *y*, 1/2 + *z*) (Fig. 1,3). In addition,*N*–H···F and C–H···F hydrogen bonds (Table 1, Fig. 3) between the helical chains and anions are also found in the crystal.

### 2. Experimental

The ligand (*N*-(pyridin-2-ylmethyl)pyridine-3-amine) was prepared according to a procedure described by Lee *et al.* (2013). Crystals of the title compound suitable for X-ray analysis were obtained by vapor diffusion of diethyl ether into a DMSO solution of the white precipitate afforded by the reaction of the ligand with silver(I) hexafluorophosphate in the molar ratio 1:1 in methanol.

### 3. Refinement

For structures of Ag^I^ coordination polymers with symmetrical dipyridyl ligands, see Lee *et al.* (2012); Leong & Vittal (2011); Park *et al*. (2010) and of Ag^I^ coordination polymers with unsymmetrical dipyridyl ligands, see: Moon & Park (2013); Zhang *et al.* (2013). For the synthesis of the ligand, see: Lee *et al.* (2013).

### Crystal data

**Table:**

[Ag(C_11_H_11_N_3_)]PF_6_	*F*(000) = 856
*M**_r_* = 438.07	*D*_x_ = 2.087 Mg m^−^^3^
Monoclinic, *P*2_1_/*n*	Mo *K*α radiation, λ = 0.71073 Å
Hall symbol: -P 2yn	Cell parameters from 5558 reflections
*a* = 10.9978 (6) Å	θ = 2.6–28.3°
*b* = 10.5081 (6) Å	µ = 1.63 mm^−^^1^
*c* = 12.7559 (7) Å	*T* = 173 K
β = 108.976 (1)°	Block, pale-yellow
*V* = 1394.04 (13) Å^3^	0.25 × 0.25 × 0.20 mm
*Z* = 4	

### Data collection

**Table:**

Bruker SMART CCD area-detector diffractometer	2740 independent reflections
Radiation source: fine-focus sealed tube	2509 reflections with *I* > 2σ(*I*)
Graphite monochromator	*R*_int_ = 0.018
φ and ω scans	θ_max_ = 26.0°, θ_min_ = 2.1°
Absorption correction: multi-scan (*SADABS*; Bruker, 2000)	*h* = −13→13
*T*_min_ = 0.687, *T*_max_ = 0.737	*k* = −12→12
7790 measured reflections	*l* = −15→7

### Refinement

**Table:**

Refinement on *F*^2^	Primary atom site location: structure-invariant direct methods
Least-squares matrix: full	Secondary atom site location: difference Fourier map
*R*[*F*^2^ > 2σ(*F*^2^)] = 0.027	Hydrogen site location: inferred from neighbouring sites
*wR*(*F*^2^) = 0.071	H-atom parameters constrained
*S* = 1.05	*w* = 1/[σ^2^(*F*_o_^2^) + (0.0342*P*)^2^ + 2.1214*P*] where *P* = (*F*_o_^2^ + 2*F*_c_^2^)/3
2740 reflections	(Δ/σ)_max_ = 0.001
199 parameters	Δρ_max_ = 0.86 e Å^−^^3^
0 restraints	Δρ_min_ = −0.44 e Å^−^^3^

### Special details

**Table:**

Geometry. All e.s.d.'s (except the e.s.d. in the dihedral angle between two l.s. planes) are estimated using the full covariance matrix. The cell e.s.d.'s are taken into account individually in the estimation of e.s.d.'s in distances, angles and torsion angles; correlations between e.s.d.'s in cell parameters are only used when they are defined by crystal symmetry. An approximate (isotropic) treatment of cell e.s.d.'s is used for estimating e.s.d.'s involving l.s. planes.
Refinement. Refinement of *F*^2^ against ALL reflections. The weighted *R*-factor *wR* and goodness of fit *S* are based on *F*^2^, conventional *R*-factors *R* are based on *F*, with *F* set to zero for negative *F*^2^. The threshold expression of *F*^2^ > σ(*F*^2^) is used only for calculating *R*-factors(gt) *etc*. and is not relevant to the choice of reflections for refinement. *R*-factors based on *F*^2^ are statistically about twice as large as those based on *F*, and *R*- factors based on ALL data will be even larger.

### Fractional atomic coordinates and isotropic or equivalent isotropic displacement parameters (Å^2^)

**Table:**

	*x*	*y*	*z*	*U*_iso_*/*U*_eq_	
Ag1	0.47207 (2)	0.61822 (2)	0.412386 (18)	0.03167 (9)	
N1	0.5497 (2)	0.4784 (2)	0.33275 (19)	0.0266 (5)	
N2	0.4485 (3)	0.2196 (3)	0.1313 (2)	0.0369 (6)	
H2	0.4838	0.1727	0.0917	0.044*	
N3	0.0973 (2)	0.2683 (2)	0.01010 (19)	0.0283 (5)	
C1	0.4751 (3)	0.3971 (3)	0.2586 (2)	0.0280 (6)	
H1	0.3845	0.4021	0.2417	0.034*	
C2	0.5256 (3)	0.3050 (3)	0.2052 (2)	0.0281 (6)	
C3	0.6588 (3)	0.3014 (3)	0.2305 (3)	0.0332 (7)	
H3	0.6972	0.2408	0.1956	0.040*	
C4	0.7343 (3)	0.3859 (3)	0.3062 (3)	0.0343 (7)	
H4	0.8252	0.3839	0.3242	0.041*	
C5	0.6775 (3)	0.4734 (3)	0.3559 (2)	0.0302 (6)	
H5	0.7303	0.5317	0.4079	0.036*	
C6	0.3137 (3)	0.2022 (3)	0.1148 (3)	0.0346 (7)	
H6A	0.3001	0.2103	0.1876	0.042*	
H6B	0.2896	0.1144	0.0880	0.042*	
C7	0.2236 (3)	0.2940 (3)	0.0343 (2)	0.0298 (6)	
C8	0.2648 (4)	0.3970 (3)	−0.0121 (3)	0.0384 (7)	
H8	0.3541	0.4145	0.0060	0.046*	
C9	0.1750 (4)	0.4743 (3)	−0.0851 (3)	0.0435 (8)	
H9	0.2021	0.5462	−0.1169	0.052*	
C10	0.0460 (4)	0.4471 (3)	−0.1120 (3)	0.0405 (8)	
H10	−0.0172	0.4982	−0.1630	0.049*	
C11	0.0118 (3)	0.3428 (3)	−0.0620 (3)	0.0358 (7)	
H11	−0.0770	0.3231	−0.0797	0.043*	
P1	0.37249 (7)	0.77956 (8)	0.11683 (7)	0.03202 (19)	
F1	0.4915 (2)	0.7903 (2)	0.22735 (18)	0.0563 (6)	
F2	0.4462 (3)	0.6767 (2)	0.0700 (2)	0.0647 (7)	
F3	0.31392 (19)	0.66683 (19)	0.17158 (17)	0.0455 (5)	
F4	0.2950 (3)	0.8808 (2)	0.1620 (3)	0.0682 (7)	
F5	0.43037 (19)	0.89076 (19)	0.06094 (19)	0.0497 (5)	
F6	0.2517 (2)	0.7676 (2)	0.00672 (18)	0.0597 (6)	

### Atomic displacement parameters (Å^2^)

**Table:**

	*U*^11^	*U*^22^	*U*^33^	*U*^12^	*U*^13^	*U*^23^
Ag1	0.03861 (15)	0.02833 (14)	0.02926 (14)	0.00868 (9)	0.01269 (10)	−0.00213 (9)
N1	0.0309 (12)	0.0256 (12)	0.0232 (12)	0.0033 (10)	0.0087 (10)	0.0017 (9)
N2	0.0353 (14)	0.0405 (15)	0.0373 (15)	−0.0029 (11)	0.0154 (12)	−0.0149 (12)
N3	0.0383 (13)	0.0253 (12)	0.0232 (12)	−0.0064 (10)	0.0127 (10)	−0.0031 (9)
C1	0.0240 (13)	0.0315 (15)	0.0279 (15)	0.0010 (11)	0.0079 (11)	−0.0011 (12)
C2	0.0312 (14)	0.0294 (14)	0.0252 (14)	0.0015 (12)	0.0109 (12)	0.0006 (11)
C3	0.0338 (15)	0.0377 (16)	0.0325 (16)	0.0065 (13)	0.0170 (13)	−0.0004 (13)
C4	0.0246 (14)	0.0443 (18)	0.0346 (17)	0.0012 (12)	0.0105 (12)	0.0030 (13)
C5	0.0322 (15)	0.0305 (15)	0.0259 (14)	−0.0044 (12)	0.0068 (12)	0.0010 (12)
C6	0.0387 (17)	0.0321 (15)	0.0307 (16)	−0.0089 (13)	0.0080 (13)	−0.0041 (12)
C7	0.0401 (16)	0.0275 (14)	0.0232 (14)	−0.0075 (12)	0.0121 (12)	−0.0054 (11)
C8	0.0502 (19)	0.0357 (17)	0.0330 (17)	−0.0148 (14)	0.0188 (15)	−0.0047 (13)
C9	0.071 (2)	0.0301 (16)	0.0350 (18)	−0.0131 (16)	0.0240 (17)	−0.0004 (13)
C10	0.062 (2)	0.0301 (16)	0.0296 (16)	0.0037 (15)	0.0158 (15)	0.0016 (13)
C11	0.0394 (16)	0.0357 (16)	0.0316 (16)	−0.0003 (14)	0.0105 (13)	−0.0017 (13)
P1	0.0292 (4)	0.0364 (4)	0.0308 (4)	−0.0005 (3)	0.0103 (3)	0.0018 (3)
F1	0.0522 (12)	0.0657 (14)	0.0409 (12)	−0.0176 (11)	0.0013 (10)	0.0084 (10)
F2	0.0786 (17)	0.0548 (14)	0.0761 (17)	0.0126 (12)	0.0465 (14)	0.0002 (12)
F3	0.0439 (11)	0.0452 (11)	0.0473 (12)	−0.0047 (9)	0.0147 (9)	0.0107 (9)
F4	0.0735 (16)	0.0467 (13)	0.105 (2)	0.0054 (11)	0.0570 (16)	−0.0062 (12)
F5	0.0384 (11)	0.0499 (12)	0.0589 (13)	−0.0044 (9)	0.0132 (10)	0.0207 (10)
F6	0.0549 (13)	0.0640 (14)	0.0469 (12)	−0.0154 (11)	−0.0018 (10)	0.0164 (11)

### Geometric parameters (Å, º)

**Table:**

Ag1—N1	2.117 (2)	C5—H5	0.9500
Ag1—N3^i^	2.130 (2)	C6—C7	1.519 (4)
Ag1—Ag1^ii^	3.2639 (5)	C6—H6A	0.9900
N1—C1	1.340 (4)	C6—H6B	0.9900
N1—C5	1.340 (4)	C7—C8	1.378 (4)
N2—C2	1.376 (4)	C8—C9	1.381 (5)
N2—C6	1.440 (4)	C8—H8	0.9500
N2—H2	0.8800	C9—C10	1.377 (5)
N3—C11	1.334 (4)	C9—H9	0.9500
N3—C7	1.348 (4)	C10—C11	1.380 (5)
N3—Ag1^iii^	2.130 (2)	C10—H10	0.9500
C1—C2	1.398 (4)	C11—H11	0.9500
C1—H1	0.9500	P1—F2	1.581 (2)
C2—C3	1.395 (4)	P1—F4	1.584 (2)
C3—C4	1.374 (4)	P1—F1	1.585 (2)
C3—H3	0.9500	P1—F6	1.594 (2)
C4—C5	1.377 (4)	P1—F5	1.604 (2)
C4—H4	0.9500	P1—F3	1.612 (2)
			
N1—Ag1—N3^i^	175.98 (9)	H6A—C6—H6B	107.4
N1—Ag1—Ag1^ii^	77.59 (6)	N3—C7—C8	121.2 (3)
N3^i^—Ag1—Ag1^ii^	105.17 (6)	N3—C7—C6	115.1 (2)
C1—N1—C5	119.2 (2)	C8—C7—C6	123.7 (3)
C1—N1—Ag1	122.10 (19)	C7—C8—C9	119.2 (3)
C5—N1—Ag1	118.67 (19)	C7—C8—H8	120.4
C2—N2—C6	123.9 (3)	C9—C8—H8	120.4
C2—N2—H2	118.1	C10—C9—C8	119.9 (3)
C6—N2—H2	118.1	C10—C9—H9	120.0
C11—N3—C7	118.9 (3)	C8—C9—H9	120.0
C11—N3—Ag1^iii^	118.4 (2)	C9—C10—C11	117.6 (3)
C7—N3—Ag1^iii^	122.7 (2)	C9—C10—H10	121.2
N1—C1—C2	122.5 (3)	C11—C10—H10	121.2
N1—C1—H1	118.8	N3—C11—C10	123.2 (3)
C2—C1—H1	118.8	N3—C11—H11	118.4
N2—C2—C3	120.4 (3)	C10—C11—H11	118.4
N2—C2—C1	122.2 (3)	F2—P1—F4	178.42 (16)
C3—C2—C1	117.4 (3)	F2—P1—F1	90.46 (15)
C4—C3—C2	119.6 (3)	F4—P1—F1	90.87 (15)
C4—C3—H3	120.2	F2—P1—F6	89.76 (15)
C2—C3—H3	120.2	F4—P1—F6	88.89 (16)
C3—C4—C5	119.6 (3)	F1—P1—F6	179.13 (13)
C3—C4—H4	120.2	F2—P1—F5	90.22 (13)
C5—C4—H4	120.2	F4—P1—F5	90.62 (13)
N1—C5—C4	121.7 (3)	F1—P1—F5	90.65 (12)
N1—C5—H5	119.1	F6—P1—F5	90.19 (12)
C4—C5—H5	119.1	F2—P1—F3	89.29 (13)
N2—C6—C7	115.6 (3)	F4—P1—F3	89.86 (12)
N2—C6—H6A	108.4	F1—P1—F3	89.85 (11)
C7—C6—H6A	108.4	F6—P1—F3	89.31 (11)
N2—C6—H6B	108.4	F5—P1—F3	179.29 (13)
C7—C6—H6B	108.4		
			
N3^i^—Ag1—N1—C1	−133.0 (12)	C3—C4—C5—N1	0.3 (5)
Ag1^ii^—Ag1—N1—C1	93.3 (2)	C2—N2—C6—C7	84.6 (4)
N3^i^—Ag1—N1—C5	45.6 (14)	C11—N3—C7—C8	1.5 (4)
Ag1^ii^—Ag1—N1—C5	−88.1 (2)	Ag1^iii^—N3—C7—C8	−175.7 (2)
C5—N1—C1—C2	1.0 (4)	C11—N3—C7—C6	−178.5 (3)
Ag1—N1—C1—C2	179.6 (2)	Ag1^iii^—N3—C7—C6	4.2 (3)
C6—N2—C2—C3	168.9 (3)	N2—C6—C7—N3	172.9 (2)
C6—N2—C2—C1	−10.4 (5)	N2—C6—C7—C8	−7.1 (4)
N1—C1—C2—N2	178.3 (3)	N3—C7—C8—C9	−0.5 (5)
N1—C1—C2—C3	−1.0 (4)	C6—C7—C8—C9	179.6 (3)
N2—C2—C3—C4	−178.7 (3)	C7—C8—C9—C10	−0.9 (5)
C1—C2—C3—C4	0.6 (4)	C8—C9—C10—C11	1.1 (5)
C2—C3—C4—C5	−0.2 (5)	C7—N3—C11—C10	−1.2 (5)
C1—N1—C5—C4	−0.6 (4)	Ag1^iii^—N3—C11—C10	176.1 (2)
Ag1—N1—C5—C4	−179.3 (2)	C9—C10—C11—N3	−0.1 (5)

Symmetry codes: (i) −*x*+1/2, *y*+1/2, −*z*+1/2; (ii) −*x*+1, −*y*+1, −*z*+1; (iii) −*x*+1/2, *y*−1/2, −*z*+1/2.

### Hydrogen-bond geometry (Å, º)

**Table:**

*D*—H···*A*	*D*—H	H···*A*	*D*···*A*	*D*—H···*A*
N2—H2···F5^iv^	0.88	2.51	3.354 (4)	160
C4—H4···F1^v^	0.95	2.52	3.336 (4)	145
C5—H5···F5^vi^	0.95	2.55	3.447 (4)	157
C5—H5···F6^vi^	0.95	2.43	3.280 (4)	149
C6—H6*A*···F3^iii^	0.99	2.54	3.467 (4)	155
C11—H11···F3^vii^	0.95	2.49	3.397 (4)	160

Symmetry codes: (iii) −*x*+1/2, *y*−1/2, −*z*+1/2; (iv) −*x*+1, −*y*+1, −*z*; (v) −*x*+3/2, *y*−1/2, −*z*+1/2; (vi) *x*+1/2, −*y*+3/2, *z*+1/2; (vii) −*x*, −*y*+1, −*z*.

## References

[bb1] Brandenburg, K. (2005). *DIAMOND* Crystal Impact GbR, Bonn, Germany.

[bb2] Bruker (2000). *SMART*, *SAINT-Plus* and *SADABS* Bruker AXS Inc., Madison, Wisconsin, USA.

[bb3] Lee, E., Ryu, H., Moon, S.-H. & Park, K.-M. (2013). *Bull. Korean Chem. Soc.* **34**, 3477–3480.

[bb4] Lee, E., Seo, J., Lee, S. S. & Park, K.-M. (2012). *Cryst. Growth Des.* **12**, 3834–3837.

[bb5] Leong, W. L. & Vittal, J. J. (2011). *Chem. Rev.* **111**, 688–764. 10.1021/cr100160e 20804195

[bb6] Moon, S.-H. & Park, K.-M. (2013). *Acta Cryst.* E**69**, m414–m415. 10.1107/S1600536813016309 PMC3772439 24046582

[bb7] Park, K.-M., Seo, J., Moon, S.-H. & Lee, S. S. (2010). *Cryst. Growth Des.* **10**, 4148–4154.

[bb8] Sheldrick, G. M. (2008). *Acta Cryst.* A**64**, 112–122. 10.1107/S0108767307043930 18156677

[bb9] Zhang, Z.-Y., Deng, Z.-P., Huo, L.-H., Zhao, H. & Gao, S. (2013). *Inorg. Chem.* **52**, 5914–5923. 10.1021/ic400055t 23634904

